# HEAT KILLED LB. PARACASEI OR CELL WALL LIPOTEICHOIC ACID AMELIORATES AGE-RELATED LEAKY GUT AND INFLAMMATION

**DOI:** 10.1093/geroni/igz038.3363

**Published:** 2019-11-08

**Authors:** Shaohua Wang, Shokouh Ahmadi, Ravinder Nagpal, Shalini Jain, Sidharth Mishra, Stephen Kritchevsky, Dalane Kitzman, Hariom Yadav

**Affiliations:** 1 Wake Forest School of Medicine, Winston-Salem, United States; 2 Wake Forest School of Medicine, Winston-Salem, North Carolina, United States; 3 Wake Forest Medical Center, Winston-Salem, North Carolina, United States

## Abstract

Increased inflammation associated with leaky gut is a major risk factor for morbidity and mortality in older adults; however, successful preventive and therapeutic strategies are not available to ameliorate these conditions. In this study, we demonstrate that a human-origin Lactobacillus paracasei D3-5 strain (D3-5), even when dead, extended life span of C. elegans. In addition, feeding D3-5 to older mice (>79 weeks) prevented high fat diet (HFD)-induced metabolic dysfunction and decreased leaky gut and inflammation, which were associated with improved physical and cognitive function. D3-5 feeding significantly increased mucin production and proportionately, the abundance of mucin-degrading bacteria Akkermansia muciniphila was also increased. Mechanistically, we show that the cell wall of D3-5 contains lipoteichoic acid (LTA), which enhanced mucin (Muc2) expression via TLR-2/p38-MAPK pathway, which in turn reduced age-related leaky gut and inflammation. This study indicates that the D3-5 and its LTA can prevent/treat age-related leaky gut and inflammation.

